# Development of talent according to Sinti and Calon Romani

**DOI:** 10.3389/fpsyg.2022.964668

**Published:** 2022-10-06

**Authors:** Jane Farias Chagas-Ferreira, Denise de Souza Fleith, Renata Muniz Prado

**Affiliations:** Department of School and Developmental Psychology, Laboratory of School Psychology, Institute of Psychology, University of Brasilia, Brasilia, Brazil

**Keywords:** Romani people, talent, education, human development, equity

## Abstract

Intercultural dialogue is a challenging demand for contemporary education, especially concerning the development of talents and potentials. This exploratory qualitative study aimed to identify factors that promote and inhibit the development of Romani talent, in Brazil, according to Sintis and Calons. Two Sinti and five Calon Romani participated in the research, aged 18 to 58 years old, males and females, with educational levels between secondary school and doctorate. A socio-demographic questionnaire and semi-structured interview protocol were used to collect data. The results indicated that talent is recognized as a set of skills associated with musicality, handicrafts, and commerce being linked to traditional Romani crafts. Internal and external inhibitory factors related to prejudice were identified through school, family and community. These barriers affected the self-esteem, personality, ambitions, and perspectives of talented young people. To achieve equity, it is necessary to provide opportunities for exchanging knowledge, practices and perspectives, in order to take into account sociocultural background of minority groups. Future research could compare nomad and non-nomad lifestyles and their impact on talent development.

## Introduction

School, as a privileged space in the education of children and young people, has been asked to reframe its curricula, practices, institutional dynamics, and internal and external interrelationships to bridge monocultural and homogenizing processes within an emancipatory and multicultural perspective of human rights (Candau, 2012, 2016; Miskovic and Curcic, 2016; Fleuri, 2018; Araújo, 2019). This scenario, as Fleuri (2018) argues, points toward the need to identify absences and emergencies of knowledge, social practices, and perspectives to promote the exchange of different knowledge available in the classroom, produced by the sociocultural subjects that are part of it (Diretoria Geral da Educacão, 2019). Recognizing, valuing, and identifying diversity does not exclude the specificities of each, which are marked by inequalities that are historically constituted by belonging to ethnic-racial groups, gender issues, or religious differences, among others (Fleuri, 2018; Araújo, 2019). These inequalities reveal recurring plots of injustices and impose effective educational actions for the promotion of basic human rights.

In this sense, talent development stands out as an innovative possibility for the expansion of inclusive education in a globalized panorama of cultural hybridizations due to its democratic, inclusive and emancipatory nature. All students should have access to opportunities, resources, and encouragement to develop their skills and aspirations through authentic, authorial, and self-fulfilling experiences (Renzulli and Reis, 2020). In this regard, services and programs that aim at talent development have to fulfill two main purposes: (a) provide opportunities for children and young people to express their potential and interests through the development of superior cognitive, leadership, artistic, scientific and socio-affective skills and (b) increase the social reserve of people who can significantly help to solve problems in contemporary society authentically and innovatively (Renzulli, 2016, 2020).

In this study, the concept of talent is used as a synonym for giftedness and high ability, being defined from the intersection of three clusters: above-average ability, creativity and task involvement (Renzulli and Reis, 2021). Above-average skill encompasses general skills (memory, attention, ease of learning) and skills associated with specific domains (plastic arts, music, dance, sports, etc.). Creativity involves divergent thinking, the ability to solve problems, and originality or productive innovation. Task involvement is about motivation and perseverance. These factors depend on individual (personological, affective, cognitive) and environmental characteristics to express or develop themselves (Renzulli, 2016).

Recent studies show the growth of research on giftedness and access to specialized educational care for gifted people in Brazil in the last decades (Alencar et al., 2019; Ferreira, 2019). Concerning ethnic minorities or traditional communities, however, there is a gap in this theme (Wechsler and Fleith, 2017; Alencar et al., 2019; Ferreira, 2019; Chagas-Ferreira, in press). Especially about specialized educational care, no records of Brazilian programs or services aimed at developing talents for the Romani were found.

It is estimated that there are more than 8 million Romani scattered across Europe, around 750,000 in the United States and more than 500,000 in Brazil. According to Brazilian census data, Roma people are present in 21 of the 27 Brazilian states and in the Federal District, in 291 camps (Batuli, 2007; Secretaria de Políticas de Promoção da Igualdade Racial, 2013). Roma people constitute one of the most numerous ethnic minorities in many countries. “The Roma people are a nation within another nation” (Batuli, 2007, p. 19). Nevertheless, in the literature about gifted education or talent development, the Romani are an invisible group even among those who are underrepresented, without even being named in these studies (Pauley and Johnstone, 2009; Siegle et al., 2016; Worrell and Dixson, 2018).

This invisibility is associated with various aspects of Roma culture and is not restricted to academic studies (Veiga and Mello, 2021). Over the centuries, the migratory processes and the dynamics of marginalization have been outlining, differentiating, and mutually distinguishing the Romani. Structural or circular nomadism, sedentarization, and linguistic hybridization are important phenomena to understand the complex Roma culture (Teixeira, 2008; Soria, 2015; Exley, 2016; Murta et al., 2016; Makarova, 2019; Silva and Figueira, 2020).

These dynamics foster endo-ethnic conflicts about the conservation and purity of the customs and traditions of the Romani people. In this search for reconstruction or reappropriation of identity, the World Congress was held in London in 1971, when unification strategies were defined as “diasporic people, possessing a common Indian root and constituting a transnational nation,” called Roma or Romani nation (Soria, 2015, p. 36). The Roma identity, as Shimura (2017, 2020) argues, is composed of multiple identity categories that reveal themselves in multifaceted experiences of “being a Romani” and “acting as a Romani.” In this sense, it is a complex task, beyond checking classic parameters indicated in the literature, such as consanguinity, knowledge and use of the ethnic language, group family union, use of typical clothing, traveling experience in tents, and Roma art (Soria, 2015; Peña, 2016; Shimura, 2017, 2020; Cairus, 2020a). In this perspective, Romani people have global, collective, or macro as well as local, individual and micro characteristics. Both conceptions are woven by belonging, definitions, and self-definitions within an exo and endo community dialogicity.

Each Roma group has semi-autonomous and coexisting cultural contours and presupposes sketching particular profiles, marked by hybridism, strained between “the feeling of ethnic belonging and the need for social integration” (Shimura, 2017, p. 29). Given these findings, Shimura (2020) proposes categories of recognition of Roma identities, constituted based on two axes: ethnic-racial (pure or authentic Romani: of father and mother; mestizos or descendants: distant/identified) and sociocultural (adopted in the family or Romani by soul). When recognizing multiple Roma identities beyond the description of subdivisions and contexts, due to their social invisibility, it is necessary to describe macro aspects of Roma identity. There are several words to designate the Romani people, the best known of which are: *gypsy*, *gitano*, *tsigane*, *bohemiens*, *zigeuner or zingars*, *egicipcian and cyan* (Soria, 2008, 2015; Secretaria de Políticas de Promoção da Igualdade Racial, 2013; Murta et al., 2016; Sierra, 2019). Along with these designations, usually pejorative, attempts are recorded to ascribe their origin to ancient Egypt or India.

The Romani people are represented in the literature by three main groups: (a) *Cale* or Calon, originating in Spain; (b) *Rom*, present in several countries of Europe, divided by nation (*natsia*): *Kalderash*, *Machuaia*, *Llovara*, *Curara and Ludar*; and (c) *Sinti* or *Manouches*, found in Germany, France, Italy, Austria, and Russia. All these groups are present in the Americas. The Calon and Sinti are subdivided into extended families or classes, camps, clans, and tents, each led by an elder. In some cases, there is a geopolitical division called a company, whose leadership corresponds to a trade association or representation to non-Romani (Soria, 2015; Murta et al., 2016; Sierra, 2019; Silva and Figueira, 2020).

In Brazil, advances in the rights of Romani people are pointed out from 1988, with the enactment of the Brazilian Constitution (Batuli, 2007; Cunha and Magano, 2019; Silva and Figueira, 2020), as well as through political activism and regulatory instruments (Dolabela and Fotta, 2021). More recently, we can highlight Brazilian publications, such as: “Guide of Public Policies for Romani” (Secretaria de Políticas de Promoção da Igualdade Racial, 2013) and “Romani People: Rights and Instruments for Their Defense” (Ministério Público Federal, 2020). These documents reveal the situation of social exclusion, whose representational violence is expressed in various media, and claim basic rights related to health, education, housing, and work. Among these are (a) guaranteeing Roma children and young people the same rights, treatment, respect, and solidarity given to non-Roma; (b) differentiated education respecting their beliefs, customs, and traditions; (c) authorship and representativeness in projects aimed at their community; (d) promotion of Roma art; and (e) ensuring that, in media and didactic material, Roma culture is respected and does not present in a positive way. It is also important to mention that the Statute of the Romani people is being processed in the Brazilian House of Legislation, supported by Bill 248/2015, whose goal is to guarantee this population equal opportunities.

This movement to recognize the rights of Roma and other ethnic minorities, especially aimed at ensuring intercultural educational policies, is the subject of recent publications in several countries: Brazil (Pinto and Oliveira, 2016; Pinel et al., 2019; Reses et al., 2019), United States (Schneeweis, 2017), Finland (Adamczewski et al., 2017), Greece (Gana et al., 2020; Kassis, 2020), Hungary (Havasi, 2019), England (Exley, 2016; D’Arcy, 2017; Parker and Levinson, 2018), Italy (Villano et al., 2017); Portugal (Magano and Mendes, 2016; Diretoria Geral da Educacão, 2019; Sierra, 2019) and the European Union (Makarova, 2019; Pop and Pop, 2019). Most of these publications advocate the need for theoretical dialogues, promotion of linguistic and communicative interdependencies, development of consistent pedagogical guidelines, greater involvement of students and their families in school projects, development of hybrid and multicultural learning ecologies, and access to opportunities to develop life skills based on community values. This scenario demonstrates the relevance of conducting research that can support public policies aimed, especially educational and health, for this population around the world.

Various facets of Roma customs foster stereotypes, especially concerning Romani women, such as hand reading (the art of divination) and fortune-telling. Music, dance, theater, pictorial art, and crafts are not only a cultural manifestation or tradition, but reveal themselves as a form of Romani subsistence and resistance (Soria, 2008, 2015, 2016; Murta et al., 2016; Peña, 2016). According to Soria (2015), Romani people “give a poetic dimension to existence, which makes them inclined to read life symbolically” (p. 126). Musicality (singing, instrument, dance) plays a fundamental role in the Romani lifestyle, assuming an ancestral cultural disposition among groups. Also, Sinti musical culture keeps many elements of its history and identity and reveals its quality through rhythmic rapidity, improvisations and virtuosity. Every repertoire is passed from father to son, from uncle to nephew through orality, experimentation, and emotion (Peña, 2016). Soria (2015) combats the idea that musicality is a natural ability, as evidenced in study by Murta et al. (2016). This understanding reduces and stigmatizes talent as being something inherited and innate, as if every Romani brought with him this brand. On the contrary, Soria (2015) argues that it is a developed skill, artistic learning that is passed from generation to generation and implies study and deliberate training. To reduce this paradoxical situation-music as a valuing and stigmatizing element of culture—she suggests carrying out diversified events that enable different cultural expressions, preferably organized by Roma representations. In her point of view, this would be a way to promote, disseminate, inspire and encourage other potentialities and characteristics of her people.

School, which could be primordial in the development of Roma potentialities, as several authors indicate, is a space seen with many caveats (Makarova and Birman, 2016; Pinto and Oliveira, 2016; Soria, 2016; Shimura, 2017; Göbel and Preusche, 2019). The families’ distrust and refusal and students’ dropout and low performance are caused by standardized, segregationist school practices, perpetuating racism, prejudices, and myths related to Romani customs and traditions. Overall, formal education, although considered fundamental to literacy (reading and writing), is still viewed with some fear due to the possible negative influence on the Roma way of life (Nicolau, 2016; Soria, 2016). On the other hand, this distancing from formal education ends up fueling a cyclical situation of marginalization and vulnerability (Tomé et al., 2016; Göbel and Preusche, 2019; Ministério Público Federal, 2020). Undoubtedly intercultural dialogue is a challenging demand for contemporary education, especially concerning the development of talents and potentials (Bennet, 2015; Tomlinson, 2017). This exploratory study aimed to identify factors that promote and inhibit the development of Roma talent according to Sintis and Calons.

## Materials and methods

### Participants

The study participants were seven Romani who stood out as eminent in their activity areas or were acknowledged for Roma leadership in Brazil, being two Sintis and five Calons. Ages ranged from 18 to 58 years. The two participants from the Sinti group did not live in a Roma camp and were university professors. All Calons lived in a Roma camp near a metropolis in the southeast of Brazil and had concluded high school. Both Sinti held an undergraduate degree (journalism and performing and plastic arts) and a graduate degree in anthropology and literature. Participants were identified by the first two letters of their group name, followed by a number and an F (female) and M (male), according to their declared gender. Despite the disparity in education, the data collected are important to understand particular ethnocultural aspects of each group, based on each participant’s peculiar perception. Four participants declared themselves social leaders or references in the community (Sinti = 1; Calon = 3), and it is important to clarify that there is no hierarchical leadership role established in a Roma community, “each head of the family has responsibility only over his family” (Si1m). Further details about the participants’ profiles are displayed in Table 1.

**Table 1:** Sociodemographic profile of participants (*n* = 7).

	Sinti 1	Sinti 2	Calon 1	Calon 2	Calon 3	Calon 4	Calon 5
Sex	M	F	M	F	M	M	M
Age	58	46	18	18	19	31	–
Education	PG *Lato Sensu*	PG *Stricto Sensu*	Primary school	Elementary education	Elementary education	Secondary school	–
Profession	Teacher	Teacher	Musician	Craftswoman	Self-employed salesperson	Welder	–
Marital status	Married	Married	Married	Married	Single	Married	–
Resident	No	No	Yes	Yes	Yes	Yes	Yes
Function or role	No	Reference	Social leader	Social leader	No	Social leader	Social leader
Talents	–	Plastic artist	Musical, multiple skills	Self-taught, craftswoman	Self-taught, learn languages	Musical	Musical, carpentry

M, males; F, females; PG, post-graduation degree.

### Instruments

Two instruments were used in this study: (a) a sociodemographic questionnaire, composed of open and closed questions about age, sex, education level, profession, and data on participation in the group’s community life; and (b) a semi-structured interview script, containing questions about the participants’ perception of how the Romani conceive talent; what characteristics the community recognizes and values in talented people; what strategies and resources are available in the community for talent development; and what challenges need to be overcome for these talents to be developed. Some examples of questions are: In your community, what skills are valued? How do these people contribute to the dynamics/routine of community life? Do people who demonstrate these skills have access to any service or support from someone in the community? How can the activities carried out at help people develop their talents?

### Procedures

Participants were contacted from an instant messaging and voice platform group (WhatsApp) through an invitation containing the objectives and procedures of the research. This group, at the time, comprised 54 people, including Roma, researchers, activists and sympathizers with the Roma cause. Those who responded positively to the invitation (*n* = 2) indicated other possible participants. All participants (*n* = 7) answered the questionnaire and signed the informed consent form, which was sent and received by e-mail or by WhatsApp. Most of the interviews were carried out through WhatsApp, by audio call, on a previously scheduled day and time. One participant preferred to respond to the survey by e-mail, due to connection problems. The interviews had the average of 35 min, ranging from 25 to 35 min.

### Data analysis

Data were analyzed using qualitative procedures based on principles of qualitative epistemology (González-Rey, 2011), which assumes: (a) the constructive-interpretative character of knowledge production, (b) research as a process of communication and dialogue, and (c) the legitimation of the singular as an instance of scientific knowledge production. Thus, from a thorough reading of the data, four indicators or areas of meaning emerged: The concept of talent linked to work and daily activities, Resources for talent development, Environmental and individual factors that act as internal and external barriers to talent development, and Strategies for removing barriers to talent development. The results of this first analysis of indicators were discussed with one of the leaders of the Roma group to align the proposed interpretations. From this perspective, the participant, as a representative of his people, is a spokesperson for himself and his social reality, ceasing to be a mere informant (Araújo et al., 2018).

## Results

As noted earlier, the Roma people do not constitute a homogeneous group. Each group has a set of cultural elements that correspond to aspects of the Roma culture in its most macro sense, while also preserving singularities that differentiate them at microsystemic levels. All participants emphasized these peculiarities at different times during the research, by all participants, being clearly explained by Si1M: “first you must understand that what you call a community (I belong to the Sinti people), does not represent the vision about talent among the other Roma groups; that is, each Roma group faces talent according to its cultural specificities.”

### Concept of talent linked to work and daily activities

With this exemption, the participants conceived talent as specific skills maintained within the family, especially in the areas of music and commerce or associated with a craft (carpenter, mason, mechanic, musician, welder, craftsman, merchant, and seamstress). Creativity was mentioned and valued as part of everyday life marked by inequalities, linked to problem-solving, and used as a way to “circumvent financial difficulties, getting money in some activity” (Si2F). Crafts were seen as the basis of the community’s income and talents as necessary for the dynamics of life in the camps and the maintenance of Roma traditions.

The perception about domains of talent was mediated by gender issues. Men engaged in carpentry, goldsmithing, stone-cutting, and the execution of musical instruments; while women engaged in handicrafts, sewing, household activities, divination arts, and singing. Ease of learning, self-study, and commercial techniques were mentioned as general characteristics. Some young people were cited because they demonstrated multiple skills, also in areas uncommon among Romani, such as language learning. Above-average talents or skills, according to the participants, would be innate (*n* = 4) or a gift (*n* = 3) that develop in a self-taught way because of interest, persistence, and individual effort. For Ca2F, women had a more articulate support network in the development of talents, as their sewing and handicraft skills were quite in demand during holiday periods. Thus, there was support for the production and the creative process.

### Resources for talent development

The perception of community resources for talent development was paradoxical. At the same time as they reported that there was no intentionally organized activity for the stimulation or promotion of talent in the local community, she was appointed as their main teacher. For Ca5M: “the teacher is the community itself, (s)he knows how to teach the other. That is how the Romani will learn from each other, even without attending school, even without having a degree.” From a very early age, according to the participants, talented Roma are more curious and demonstrate their interests and abilities through personal effort, self-education, or by observing adults and young people. They are more advanced in their areas of interest when compared to other children. For Ca5M, the Roma family, despite identifying these characteristics, generally cannot help in this development, due to financial issues or due to the low level of education of their parents. In this regard, the family would need help and guidance.

Regarding the factors that promote the development of talents or resources available in the community, there was a controversial perception among the participants. Some pointed out that the necessary resources were present within the community (*n* = 2), being an issue inherent to the maintenance of their traditions. This understanding can be summed up by the perception of Ca5M: “one helps the other, one gradually teaches things to the other.” The younger ones (Ca1M, Ca2F, and Ca3M), however, highlighted factors that acted as barriers, such as poor access to formal education or schooling; little financial incentive; lack of proper place for artisanal production and specific training courses (music, sales techniques); and lack of professionals or mentors from the community itself.

### Environmental and individual factors that act as internal and external barriers to talent development

Low education level and little understanding of the rights and duties of the Roma people were identified as factors that harm the recognition of potential and talents. This scenario was aggravated by prejudice and affirmative actions that did not meet the specificities of the Roma lifestyle. Prejudice was perceived through internal and external pathways. As an internal element, those who sought knowledge, education, or improvement were recriminated by the judgment that they abandoned the Roma culture. On the other hand, in society in general, and especially in school, they suffered from the stereotypes and discrimination related to the Roma lifestyle and values.

There was a consensus among the participants that: (a) music was the artistic skill the Romani most valued and most widely recognized, with several famous musicians being named nationally and internationally; and (b) prejudice was the biggest barrier to be overcome, as it exerted a negative influence on the identity, personality, and subjectivity of the young Romani. This process would entail consequences in the socio-emotional adjustment which influenced the development of talents, such as low self-esteem, shyness, disinterest in improvement outside the community, and little ambition to develop potential.

### Strategies for removing barriers to talent development

To break through these barriers greater awareness of the state, society, and the community itself was advocated. The Roma community should have greater access to knowledge on the subject and be challenged to *get out of its closed world* and seek ways to get education, as Ca5M points out: “the Romani always encourages the other not to study, to no longer seek to get training with those outside.” On the other hand, it was recognized that *the bad things experienced* ended up limiting the demand for external help or for means to improve talent and that specific public education policies for Roma were needed. Without this, as Si2F argued, “the community will continue to waste its talents by not having the basic conditions to identify its potentialities or promote its improvement.” Without opportunities within the community (appreciation, affection, stimulation, adequate space, specific projects, tools, and material resources) and help from outside (teachers, instructors, and courses) talents will not find fertile ground to develop, concluded Si2F.

As a future perspective for the development of Roma potentialities, two aspects to be considered were pointed out: (a) planning events and projects aimed at mapping and attending talents, as a way to strengthen and empower the Roma community; and (b) access to formal education that has an emancipating role in the current poverty situation of some communities, without them having to give up their customs, values, and traditions.

## Discussion

The results indicated that the process of talent development among the Romani is dependent on broader social and educational inclusion measures. Without having their rights guaranteed, as a transnational organization with dynamics, beliefs, and values distinct from the surrounding community, their potential tends to remain neglected and deferred. This set of measures and strategies lacks multilevel—social, institutional, and material—inclusion plans, as stated by Murta et al. (2016). These measures would start from objective, measurable, and systematically evaluated mappings, allowing equal access to opportunities for employment, education, health, security, justice, and social participation (Batuli, 2007; Diretoria Geral da Educacão, 2019; Göbel and Preusche, 2019; Ministério Público Federal, 2020).

The talents and potentialities recognized were strictly related to the crafts and skills already described in the literature, as characteristic of Roma groups, such as trade, music, goldsmithing, and negotiation (Soria, 2015, 2016; Peña, 2016; Ministério Público Federal, 2020), without mentioning palmistry practices. Creativity was associated with the ability to solve everyday problems, especially related to financial adversity. Ease of learning and self-education were two other individual characteristics of talented people, the domain of talent being mediated by the division of productive activities and gender. These results corroborate findings from previous studies that talent development in underrepresented groups is closely related to aspects of survival and resistance of cultural identities and individual traits (Siegle et al., 2016; Cairus, 2020b). The role and support of experts and mentors external to the community were also evidenced, as a way to leverage performances and production. Mentoring as a personal relationship between a professional and the mentee is considered an effective intervention which through modeling, instruction and provision of opportunities can minimize the impact of social pressure in the Roma talent development process (Grassinger and Ziegler, 2010).

Prejudice was the main barrier identified, being experienced at school and in the surrounding community due to exclusionary and stigmatizing processes (Havasi, 2019). As with other minorities, this situation can lead to the denial of Roma identity, which can further reinforce social invisibility and waste of talents (Siegle et al., 2016; Silva and Figueira, 2020). These dynamics seem to naturalize school failure, poverty, and the precarious state of children and youth from minority groups while signaling the scarce access to opportunities for success and genuine expression of their abilities (Nicolau, 2016; Shaw et al., 2016; Tomé et al., 2016).

To have their talents recognized, the young Romani would need to break with internal and external social pressures. Concerning internal pressures, fear, recrimination, and distrust were evidenced in that their cultural roots and identity are tainted and abandoned due to education processes or wide access to technologies. At the same time, they need to respond to external pressures that disregard their linguistic peculiarities, ways of thinking, learning styles, values, customs, and knowledge. These results corroborate the literature regarding the endogenous and exogenous pressures children and young talents from traditional communities (Indigenous, Roma, quilombolas, among others) are subject to. As an example of endogenous pressure, we can cite family practices. According to an interview by Cairus (2020a), Mio Vacite, an excellent violinist and Roma activist, had to study hidden from his family. On one occasion, his instrument was broken in a parental attempt to prevent him from continuing the development of his musical talent.

As examples of exogenous pressure, the school context and gaps in legislation can act as important inhibitory factors or barriers. In a study involving natives of the *Wapixana* people (Chagas-Ferreira, 2021), punishment of an indigenous child for drawing on a school table was reported, without considering the conditions in which this type of work was produced in their community or the quality of their drawing. In a case study with an adolescent from a rural settlement, Ferreira (2019) found that the teachers ignored his talents and potential. What is more, the mother had to answer for the boy’s performance, not due to learning issues, but to the fact that the boy arrived dirty of earth at school. Marcos Terena, one of the great Indigenous leaders of the world, had his dream of becoming a commercial pilot prevented, after having fulfilled all the requirements with excellence and in conditions of extreme inequality, due to legislation that declared him “incapable” (Feijó and Terena, 1994). Despite the expansion of quota systems in Brazil, there is still a need for judicialization so that minority rights are guaranteed.

The results of this study indicate the need for greater investment in the awareness of community agents and in the training of teachers/tutors/advisors who can work in intercultural schools and in the consolidation of a democratic, emancipatory and anti-racist education (Cunha and Magano, 2019). Breaking the cycle of school failure and the high school dropout rates of Roma children and young people requires structural and functional changes in the provision of formal education (Tomé et al., 2016). This implies a change oriented towards school success, potential and talents. This process mobilizes individual capacities, competencies, and experiences, based on a logic of assets instead of deficits (Chagas-Ferreira, 2016).

In this perspective, enrichment activities can leverage the empowerment of students and the development of potentialities by mobilizing traditional knowledge, social practice, and individual capacities to propose authentic solutions to community problems (Renzulli, 2016; Renzulli and Reis, 2020, 2021). These activities can be planned and implemented in the school or the community, aiming to put students in contact with different domains of human knowledge, working on scientific, performance, artistic, entrepreneurial, research projects, or offering services based on their styles and ways of life. This exploratory study pointed to gaps and emergencies in the promotion and development of Roma talents. On the other hand, it also showed that we experience a privileged historical moment when the literature reveals greater sensitization, awareness, and mobilization towards guaranteeing the rights of the Romani people and ethnic minorities and in the formulation of inclusive emancipatory policies around the world.

### Final considerations

The dominant logic in Western school education processes is still standardizing, homogenizing, and monocultural. Advancing in an intercultural perspective requires the promotion of a success-oriented ecology of knowledge, centered on nonconformist and emancipatory subjectivities, and the development of potentialities and talents. Giving access to opportunities that take into account the socio-cultural specificities of minority groups and provide opportunities for the exchange of different knowledge, practices, and perspectives seems to be a necessary and structural condition in the construction of social justice.

This study has some limitations. The sample size and the heterogeneity of the participants, in addition to being a convenience sample, are insufficient to explain the development of Roma talent on a scale. The use of interviews with open-ended questions also limits the generalization of the results. For future research, it is recommended to deepen aspects related to the development of Roma talents associated with gender differences, domains of talent, and levels of education, with data collection from multiple sources of information from the Roma community, in addition to ethnographic studies. It is also relevant to conduct a comparative study with groups in different situations of nomadism and sedentary lifestyle to verify perceptions and practices concerning talent development.

The findings of this study stress the need for a more flexible identification process considering the characteristics of the Romani, as suggested for other under-represented groups as well (El Khoury and Al-Hroub, 2018; Worrell and Dixson, 2018, 2022; Al-Hroub, 2022; Renzulli and Brandon, 2022). In addition, it is imperative to offer professional development opportunities in the field of giftedness for teachers of Roma students.

## Data availability statement

 The datasets presented in this article are not readily available because the authors do not have the participants’ authorization for sharing the data. Requests should be directed to janefc@unb.br.

## Ethics statement

This study was carried out in accordance with recommendations of the Helsinki Declaration. We collected written informed consent from the participants.

## Author contributions

JC-F was responsible for designing the study, collecting, analyzing, and interpreting the data, and writing the draft. DF contributed with critical comments throughout the different versions of the draft, and contributed to the discussion review, and writing of the final version of the manuscript. RP provided critical comments throughout the different versions of the draft, contributed to the introduction review, as well as to the writing of the final version of the manuscript. All authors contributed to the article and approved the submitted version.

## Conflict of interest

The authors declare that the research was conducted in the absence of any commercial or financial relationships that could be construed as a potential conflict of interest.

## Publisher’s note

All claims expressed in this article are solely those of the authors and do not necessarily represent those of their affiliated organizations, or those of the publisher, the editors and the reviewers. Any product that may be evaluated in this article, or claim that may be made by its manufacturer, is not guaranteed or endorsed by the publisher.

## References

[ref1] Adamczewski J. Akademi A. Mickiewicz A. (2017). The multicultural school. Analysis of challenges and opportunities based on examples from vasa Övningsskola. Biuletyn Historii Wychowania 36, 77–84. doi: 10.14746/bhw.2017.36.5

[ref2] Alencar E. M. L. S. Fleith D. S. Carneiro L. B. (2019). “Gifted education in Brazil: historical background, current practices, and research trends,” in The Sage handbook of Gifted and Talented Education. eds. Wallace B. Sisk D. A. Senior J. (Thousand Oaks: SAGE), 432–444. doi: 10.4135/9781526463074.n36

[ref3] Al-Hroub A. (2022). Gifted education in Lebanon: re-examining the role of educational and learning capitals. Cog. Educ. 9, 1–19. doi: 10.1080/2331186X.2022.2073644

[ref4] Araújo R. S. (2019). Resenha crítica: Educação intercultural e formação de professores critical review: intercultural education and techers’ professional development. Revista Interinstitucional Artes de Educar 4, 701–706. doi: 10.12957/riae.2018.40850

[ref5] Araújo C. M. Oliveira M. C. S. L. Rossato M. (2018). O sujeito na pesquisa qualitativa: Desafios da investigação dos processos de desenvolvimento [the individual in qualitative research: challenges of the development processes investigation]. Psicologia: Teoria e Pesquisa 33, 1–7. doi: 10.1590/0102.3772e33316

[ref6] Batuli M. S. (2007). Povo cigano: O direito em suas mãos [Romani people: the right in your hands]. Secretaria Especial dos Direitos Humanos. http://static.paraiba.pb.gov.br/2016/05/cartilha-ciganos.pdf

[ref7] Bennet J. M. (ed.) (2015). The SAGE Encyclopedia of Intercultural Competence, vol. 1. Thousand Oaks: SAGE.

[ref8] Cairus B. G. (2020a). “Mio Vacite: Anticiganismo, transnacionalismo e a formação da União Cigana do Brasil [Mio Vacite: anti-Roma, transnationalism and the formation of the Brazilian Roma union],” in Povos ciganos: Direitos e instrumentos para sua defesa. ed. Federal M. P. (Brasilia: Ministério Público Federal), 162–203. http://www.mpf.mp.br/pgr/documentos/maio_cigano_coletanea_versao_final.pdf

[ref9] Cairus B. G. (2020b). A construção das identidades ciganas no Brasil [The construction of Roma identities in Brazil]. Revista USP 117, 1–17. http://jornal.usp.br/especial/revista-usp-117-a-construcao-das-identidades-ciganas-no-brasil/

[ref10] Candau V. M. F. (2012). Diferenças culturais, interculturalidade e educação em direitos humanos [Cultural differences, interculturality and human rights education]. Educação e Sociedade 33, 235–250. doi: 10.1590/S0101-73302012000100015

[ref11] Candau V. M. F. (2016). “Ideias-força” do pensamento de Boaventura Sousa Santos e a educação intercultural [“force-ideas” in Boaventura Sousa Santos’ thinking and intercultural education]. Educação em Revista 32, 15–34. doi: 10.1590/0102-4698140011

[ref12] Chagas-Ferreira J. F. (2016). “Psicologia escolar e desenvolvimento humano: Articulação de saberes para a promoção do sucesso escolar [School psychology and human development: Articulation of knowledge to promote school success],” in *Psicologia Escolar Crítica: Teoria e Prática nos Contextos Educacionais* [*Critical School Psychology: Theory and Practice in Educational Contexts*]. eds. Dazzani M. Souza V. L. T. (Campinas: Alínea), 117–136.

[ref13] Chagas-Ferreira J. F. (2021). *Um Estudo de Caso Sobre o Talento Entre os Wapixana* [*A Case Study on Talent Among the Wapixana; Manuscript Submitted for Publication*]. Brasilia: Institute of Psychology, University of Brasilia.

[ref14] Chagas-Ferreira J. F. (in press). *O talento entre os Yuhup: um estudo de Caso* [*Talent Among the Yuhup: A Case Study*] Psicologia em Revista.

[ref15] Cunha J. R. Magano O. (2019). Ciganas e ciganos no Brasil e Portugal: Uma análise comparativa acerca dos processos de integração e construção de políticas sociais [female and male Roma in Brazil and Portugal: A comparative analysis of the processes of integration and construction of social policies]. Rev. Anthropol. 30, 251–280. doi: 10.51359/2525-5223.2019.244023

[ref16] D’Arcy K. (2017). Using counter-stories to challenge stock stories about traveller families. Race Ethn. Educ. 20, 636–649. doi: 10.1080/13613324.2016.1191701

[ref17] Diretoria Geral da Educacão. (2019). Promover a inclusão e o sucesso educativo das comunidades ciganas: Guião Para as escolas [promoting the inclusion and educational success of Roma communities: guide for schools]. https://www.dge.mec.pt/sites/default/files/ECidadania/Educacao_Intercultural/documentos/guiao_comunidades_ciganas.pdf

[ref18] Dolabela H. Fotta M. (2021). “Ciganos as a traditional people: Romanies and the politics of recognition in Brazil,” in Ethnopolitics: Advance Online Publication, 1–20. doi: 10.1080/17449057.2021.2008671

[ref19] El Khoury S. Al-Hroub A. (2018). Gifted education in Lebanese schools: integrating theory, research, and practice. Springer. doi: 10.1007/978-3-319-78592-9

[ref20] Exley S. (2016). “Inside and outside the school gates: impacts of poverty on children’s education,” in Improving Children’s Life Chances. ed. Tucker J. (London: Child Poverty Action Group), 1–8. https://www.researchgate.net/publication/316097404

[ref21] Feijó A. Terena M. (1994). O índio aviador [the indian aviator]. Moderna.

[ref22] Ferreira U. L. (2019). *O talento no assentamento rural: O estudo de Caso de um adolescente superdotado* [*talent in the rural settlement: The Case Study of a Gifted Teenager Unpublished Master’s Thesis*]. Brasilia: University of Brasilia. https://repositorio.unb.br/handle/10482/37657

[ref23] Fleuri R. M. (2018). *Educação Intercultural e Formação de Professores* [*Intercultural Education and Teachers’ Professional Development*]. João Pessoa: Editora do CCTA.

[ref24] Gana E. Stathopoulou C. Govaris C. (2020). “Expanded pedagogical spaces: enhancing Roma students’ involvement in school,” in *Language Diversity in Greece*. eds. Skourtou E. Kourtis-Kazoullis V. Aravossitas T. Trifonas P. P. (Cham: Springer), 169–181. doi: 10.1007/978-3-030-28396-4_13

[ref25] Göbel K. Preusche Z. M. (2019). Emotional school engagement among minority youth: the relevance of cultural identity, perceived discrimination, and perceived support. Intercult. Educ. 30, 547–563. doi: 10.1080/14675986.2019.1616263

[ref26] González-Rey F. L. (2011). *Pesquisa Qualitativa em Psicologia: Caminhos e Desafios* [*Qualitative Research in Psychology: Paths and Challenges*]. São Paulo: Cengage Learning.

[ref27] Grassinger P. M. Ziegler A. (2010). Mentoring the gifted: A conceptual analysis. High Abil. Stud. 21, 27–46. doi: 10.1080/13598139.2010.488087

[ref28] Havasi V. (2019). Social problems and economic performance: social innovations in the Hungarian child protection system. J. Econ. Lit. 15, 11–22. doi: 10.18096/TMP.2019.02.02

[ref29] Kassis D. (2020). “Underachievement of Roma children in Greece,” in *Language diversity in Greece*. eds. Skourtou E. Kourtis-Kazoullis V. Aravossitas T. Trifonas P. P. (Cham: Springer), 183–192. doi: 10.1007/978-3-030-28396-4_14

[ref30] Magano O. Mendes M. M. (2016). Constrangimentos e oportunidades Para a continuidade e sucesso das pessoas ciganas [constraints and opportunities for the continuity and success of Roma people]. Configurações: Revista de. Sociologia 18, 8–26. doi: 10.4000/configuracoes.3546

[ref31] Makarova E. (2019). Acculturation and school adjustment of minority students: school and family-related factors. Intercult. Educ. 30, 445–447. doi: 10.1080/14675986.2019.1643559

[ref32] Makarova E. Birman D. (2016). Minority students’ psychological adjustment in the school context: an integrative review of qualitative research on acculturation. Intercult. Educ. 27, 1–21. doi: 10.1080/14675986.2016.1144382

[ref33] Ministério Público Federal. (ed.) (2020). *Povos Ciganos: Direitos e Instrumentos Para sua Defesa* [Romani Peoples: Rights and Instruments for Their Defense]. Brasilia: Ministério Público Federal. http://www.mpf.mp.br/pgr/documentos/ maio_cigano_coletanea_versao_final.pdf

[ref34] Miskovic M. Curcic S. (2016). Beyond inclusion: reconsidering policies, curriculum, and pedagogy for Roma students. Int. J. Multicult. Educ. 18, 1–14. doi: 10.18251/ijme.v18i2.1051

[ref35] Murta J. B. Santos P. C. Silva A. M. M. (2016). A invisibilidade cigana no Brasil: que ações podem ser desenvolvidas pelo profissional de serviço social [Roma invisibility in Brazil: what actions can be developed by the social service professional]? Revista Digital de Ciencias Sociales III, 205–226. https://revistas.uncu.edu.ar/ojs/index.php/millca-digital/article/view/768

[ref36] Nicolau L. F. (2016). Complexidades no percurso escolar das crianças ciganas: Relatos de pais e professores [complexities in the school career of Roma children: reports from parents and teachers]. Configurações: Revista de. Sociologia 18, 105–121. doi: 10.4000/configuracoes.3682

[ref37] Parker R. Levinson M. P. (2018). Student behavior, motivation and the potential of attachment-aware schools to redefine the landscape. Br. Educ. Res. J. 44, 875–896. doi: 10.1002/berj.3473

[ref38] Pauley G. Johnstone K. (2009). Addressing under-representation of student populations in gifted programs. http://www.k12.wa.us/sites/default/files/public/highlycapable/pubdocs/2010/underrepresentationgiftedprograms.pdf

[ref39] Peña I. O. (2016). Exploring the long journey of Sinti resistance and identities with musical narratives. Soc. Just. Persp. http://hdl.handle.net/2105/37337

[ref40] Pinel W. R. Perpétuo L. D. Reses E. S. (2019). Educação, territorialidade e luta cigana: um estudo de Caso do povo Calon no Distrito Federal [education, territoriality and Roma struggle: A case study of the Calon people in the Federal District]. Poiésis Pedagógica 17, 139–150. doi: 10.5216/rpp.v17il.59056

[ref41] Pinto A. K. P. Oliveira I. M. (2016). Ciganos na escola: Desafios e potencialidades [Romani perople at school: challenges and potential]. J. Res. Spec. Educ. Needs 16, 295–298. doi: 10.1111/1471-3802.12151

[ref42] Pop L. Pop I. (2019). Marginal multilingualism and its potential in fighting the xenophobia/romaphobia. J. Ident. Migrat. Stud. 13, 81–98. http://www.e-migration.ro/jims/Vol13_No1_2019/JIMS_Vol13_No1_2019_ pp_81_98_POP.pdf

[ref43] Renzulli J. S. (2016). “Reexamining the role of gifted education and talent development for the 21st century,” in Reflections on Gifted Education. ed. Reis S. M. (Waco: Prufrock Press), 31–51.

[ref44] Renzulli J. S. (2020). Promoting social capital by expanding the conception of giftedness. Talent 10, 2–20. doi: 10.46893/talent.757477

[ref45] Renzulli J. S. Brandon L. E. (2022). “Considerations for increasing access to talent development programs for students from low-income and minority groups,” in Education Inclusive in High Capacities: Arguments and Perspectives. eds. Rocha A. Perales R. Ziegler A. Renzulli J. S. Gagné F. Pfeiffer, S. I. (Porto: Aneis), 33–50.

[ref46] Renzulli J. S. Reis S. M. (2020). “The design of the three rings of gifted and the school enrichment model: A talent development approach for all students,” in Conceptual Frameworks for Giftedness and Talent Development: Enduring Theories and Comprehensive Models in Gifted Education. eds. Cross T. L. Olszewski-Kubilius P. (Waco: Prufrock Press), 145–180.

[ref47] Renzulli J. S. Reis S. M. (2021). “The three-ring conception of giftedness: A change in direction from being gifted to the development of gifted behaviors,” in Conceptions of Giftedness and Talent. eds. Sternberg R. J. Ambrose D. (New York: Palgrave Macmillan), 335–356. doi: 10.1007/978-3-030-56869-6_19

[ref48] Reses E. S. Pinel W. R. Perpetuo L. D. (2019). Entre tendas e escolas: Desafios Para a educação formal do povo cigano no Distrito Federal [between tents and schools: challenges for the formal education of the Romani people in the Federal District]. Plurais: Revista Multidisciplinar 4, 189–202. doi: 10.29378/plurais.2447-9373.2019.v4.n1.189-202

[ref49] Schneeweis A. (2017). The imagined backward and downtrodden other: contemporary American news coverage of the Roma/gypsy. Journal. Stud. 19, 2187–2206. doi: 10.1080/1461670x.2017.1331708

[ref50] Secretaria de Políticas de Promoção da Igualdade Racial. (2013). Guia de políticas públicas para povos ciganos [Public policy guide for Romani people]. https://bibliotecadigital.mdh.gov.br/ jspui/handle/192/309

[ref51] Shaw B. Bernardes E. Trethewey A. Menzies L. (2016). Special educational needs and their links to poverty. https://www.jrf.org.uk/report/special-educational-needs-and-their-links-poverty

[ref52] Shimura I. (2017). Ser cigano: Identidade étnica em um acampamento Calon itinerante [being a Romani: ethnic identity in a traveling Calon camp]. Amazon.

[ref53] Shimura I. (2020). “A ideia de ciganidade Como chave Para o reconhecimento da pluralidade cigana no Brasil [the idea of gypsy as a key to the recognition of Roma plurality in Brazil],” in Povos ciganos: Direitos e Instrumentos para sua defesa. ed. Federal M. P., 45–77. http://www.mpf.mp.br/pgr/documentos/ maio_cigano_coletanea_versao_final.pdf

[ref54] Siegle D. Gubbins J. E. O’Rouke P. Langley S. D. Mun R. U. Luria S. R.. (2016). Barriers to underserved students’ participation in gifted programs and possible solutions. J. Educ. Gift. 39, 103–131. doi: 10.1177/0162353216640930

[ref55] Sierra M. (2019). Creating Romanestan: A place to be a gypsy in post-nazi Europe. Eur. Hist. Q. 49, 272–292. doi: 10.1177/0265691419836909

[ref56] Silva P. C. S. Figueira L. E. V. (2020). Direitos, identidade e povos ciganos: um estudo sobre as Fronteiras dos processos de normatização da ciganidade no Brasil [rights, identity and the Romani people: A study on the frontiers of gypsy normatization processes in Brazil]. Revista Direitos Culturais 15, 159–200. doi: 10.20912/rdc.v15i35.3280

[ref57] Soria A. P. C. B. (2008). *Entre a dor de ser “cigano” e o orgulho de ser romà: Aproximação à literatura romani e a autorepresentação dos romà em duas obras de Jorge Nedich* [*Between the Pain of Being a “Gypsy” and the Pride of Being Roma: Approach to Romani Literature and the Self-Representation of Roma in Two Works by Jorge Nedich; Unpublished Master’s Thesis*]. Brasilia: University of Brasilia. http://repositorio.unb.br/handle/10482/1365.

[ref58] Soria A. P. C. B. (2015). *“Juncos ao vento”: Literatura e identidade romani* (*cigana*)*: El alma de los parias*, *de Jorge Nedich* [*“juncos in the wind”: Romani* (*gypsy*) *Literature and Identity: El alma de los parias*, *by Jorge Nedic; Unpublished Doctoral Dissertation*]. Brasilia: University of Brasilia. https://repositorio.unb.br/handle/10482/19111.

[ref59] Soria P. (2016). Nas tessituras da emergente literatura Romani (cigana): Subalternidade, gênero e identidade em questão [in the intrincacies of the emerging Romani (gypsy) literature: Subalternity, gender and identity in question]. Revista Eletrônica Literatura e Autoritarismo: Narrativa Testemunhal e Relações Históricas 27, 21–31. doi: 10.5902/1679849X22562

[ref60] Teixeira R. C. (2008). História dos ciganos no Brasil [History of Romani in Brazil]. Núcleo de estudos ciganos. http://www.dhnet.org.br/direitos/sos/ciganos/a_pdf/rct_historiaciganos brasil2008.pdf

[ref61] Tomé M. C. Carvalho A. Sousa J. Saraiva D. Domingues A. Olivera M. F. (2016). O sucesso escolar dos alunos de etnia cigana: Desafios emergentes. O Caso dos alunos do agrupamento de escolas Infante D. Henrique [school success of Roma students: emerging challenges. The case of the students of the Infante D. Henrique school group]. Configurações: Revista de. Sociologia 18, 87–104. doi: 10.4000/configuracoes.3659

[ref62] Tomlinson S. (2017). A Sociology of Special and Inclusive Education Exploring the Manufacture of Inability. New York: Routledge. doi: 10.4324/9781315646237

[ref63] Veiga F. B. Mello M. A. S. (2021). “Adro dos ciganos: Quadros sociais da memória e políticas do reconhecimento no espaço público carioca [Adro of Romani: social frameworks of memory and politics of recognition in the public space of Rio de Janeiro],” in Pesquisa empírica aplicada ao direito: Perspectivas teóricas e metodológicas de reconhecimento de direitos. eds. Miranda A. P. M. Oliveira I. M. (Rio de Janeiro: Telha), 363–400. https://editoratelha.com.br/product/pesquisa-empirica-aplicada-ao-direito-perspectivas-teoricas-e-metodologicas-sobre-o-reconhecimento-de-direitos/

[ref64] Villano P. Fontanella L. Fontanella S. Donatoda M. D. (2017). Stereotyping Roma people in Italy: IRT models for ambivalent prejudice measurement. Int. J. Intercult. Relat. 57, 30–41. doi: 10.1016/j.ijintrel.2017.01.003

[ref65] Wechsler S. M. Fleith D. S. (2017). The scenario of gifted education in Brazil. Cogent Educ. 4:1332812. doi: 10.1080/2331186X.2017.1332812

[ref66] Worrell F. C. Dixson D. D. (2018). “Recruiting and retaining underrepresented gifted students,” in *Handbook of Giftedness in Children*. *Psychoeducational Theory*, *Research*, *and Best Practices*. ed. Pfeiffer S. I. (Cham: Springer), 209–226. doi: 10.1007/978-3-319-77004-8_13

[ref67] Worrell F. C. Dixson D. D. (2022). Achieving equity in gifted education: ideas and issues. Gifted Child Q. 66, 79–81. doi: 10.1177/00169862211068551

